# Insulin, IGF-I, and lactoferrin concentrations and yields and their associations with other components within colostrum, transition, and whole milk of primiparous and multiparous Holstein cattle

**DOI:** 10.3168/jdsc.2024-0572

**Published:** 2024-07-14

**Authors:** A.J. Fischer-Tlustos, S.L. Cartwright, K.S. Hare, D.J. Innes, J.P. Cant, M. Tortades, F. Fabregas, A. Aris, E. Garcia-Fruitos, M.A. Steele

**Affiliations:** 1 Department of Animal Biosciences, Animal Science and Nutrition, University of Guelph, Guelph, ON, Canada N1G 1Y2; 2 Department of Animal and Poultry Science, University of Saskatchewan, Saskatoon, SK, Canada S7N 5A8; 3 Ruminant Production Programme, Institute of Agrifood Research and Technology, Caldes de Montbui, 08173, Spain

## Abstract

The primary objective of the study was to characterize concentrations and yields of lactoferrin (LF), insulin, and IGF-I in colostrum, transition milk (TM), and whole milk (WM) of multiparous (MP) and primiparous (PP) cows. A secondary objective was to determine associations between colostrum and TM components (fat, protein, lactose), IgG, and bioactive compounds (oligosaccharides, LF, insulin, IGF-I; defined as compounds present in micro quantities that stimulate physiological responses systemically or locally within the neonate). Holstein cows (10 MP and 10 PP) were assigned to the study at calving and colostrum was collected 5.3 ± 0.7 h (mean ± SE) after calving, followed by twice daily sampling of milkings 2–5 (TM) and milking 12 (WM). Colostrum, TM, and WM samples were analyzed by commercial ELISA to determine concentrations of LF, insulin, and IGF-I. Concentrations of insulin, IGF-I, and LF were greatest in colostrum compared with all other milkings. Similarly, IGF-I and LF yields were greatest in colostrum, whereas insulin yield was greatest in colostrum and milking 2 compared with milking 4, 5, and 12. Primiparous cows had greater insulin and lower LF concentrations in colostrum compared with MP cows. Additionally, lower LF concentrations in PP cows persisted through milkings 2 and 3. The majority of associations between components and bioactive compounds in colostrum were positive; however, correlations were different between PP and MP cows. Specifically, there were a greater number of strong (ρ > 0.80) correlations between components and IgG in PP cows. In contrast, MP cows demonstrated a greater number of strong correlations among bioactive compounds. To the authors' knowledge, this study is the first to characterize the yields of IGF-I, LF, and insulin during the colostral (collection within 6 h) and TM period in both MP and PP cows. The study findings indicate that parity influences the levels of LF and insulin, but not IGF-I. Our study also uniquely demonstrates associations between early lactation components and bioactive compounds, which are not consistent among parities.

## Highlights

• Insulin and IGF-I gradually decrease from colostrum to whole milk, whereas LF yield is similar in TM and whole milk.
• PP cows produced greater insulin and lower LF in colostrum than MP cows.
• Correlations between colostrum composition are not consistent among parities.

## Graphical Abstract

**Figure:**
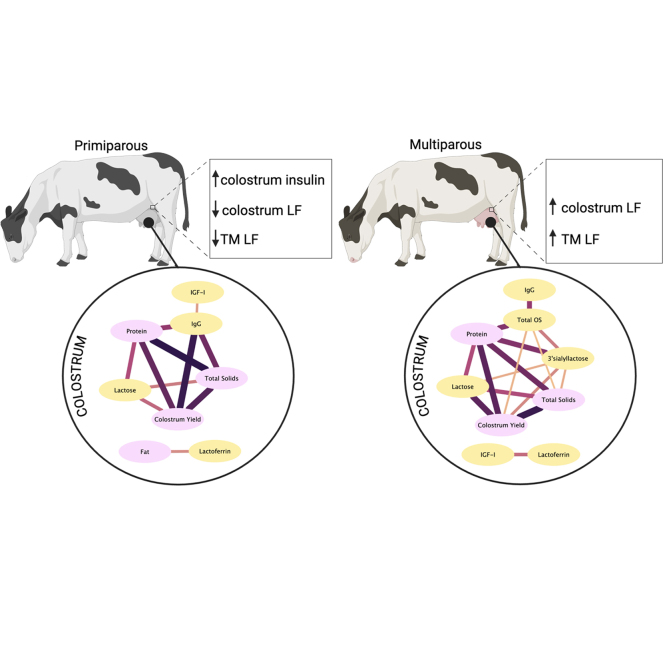
**Summary:** Multiparous (MP; n = 10) and primiparous (PP; n = 10) cows were followed from 1 to 7 days in milk to evaluate the levels of insulin, insulin-like growth factor-I (IGF-I), and lactoferrin (LF) in colostrum (milking [M]1), transition milk (TM; M2-5), and whole milk (M12). In colostrum, PP cows had higher insulin and lower LF than MP cows, with lower LF persisting into the TM period. Spearman's rank correlation analyses between components (pink nodes) and bioactives (yellow nodes) demonstrated strong (ρ > 0.80; darker, thicker edges) positive associations between colostrum IgG and colostrum yield, total solids, and protein yield in PP, but not MP, cows. In contrast, MP cows demonstrated a greater number of strong correlations between bioactives in colostrum, namely between IGF-I and LF, and oligosaccharides (OS) and IgG. Figure was created with BioRender.com; association maps with Cytoscape.org.

Over recent decades, colostrum research has largely focused on strategies to maximize IgG mass due to its importance in ensuring transfer of passive immunity (**TPI**) in the neonatal calf (Morrill et al., 2012). Simultaneously, additional research, albeit lesser in number, was published characterizing the myriad of bioactive components in bovine colostrum aside from IgG (McGrath et al., 2016; Tacoma et al., 2017). This knowledge has subsequently led to a greater appreciation for the wide array of developmental, immunomodulatory, and health-promoting properties of colostrum, rather than solely providing TPI via high concentrations of IgG. The number of colostral bioactive compounds and their potential impact on the immunologically and metabolically naïve neonatal calf is immense and has been reviewed extensively (McGrath et al., 2016; Fischer-Tlustos et al., 2021). In terms of antimicrobial compounds, colostrum is rich in lactoferrin (**LF**; Kehoe et al., 2007), which can lessen calf morbidity and improve recovery, as well as calf growth, when supplemented preweaning (Robblee et al., 2003). Similarly, bovine colostrum is also rich in peptide hormones, containing ∼65 and >300 times greater levels of insulin and IGF-I than whole milk (**WM**), respectively (Blum and Hammon, 2000). In contrast to LF, the benefits of these peptide hormones are largely attributed to local effects on neonatal gastrointestinal development and enhancing intestinal capacity for glucose absorption (Hammon et al., 2020).

The wide spectrum of bioactive compounds in maternal colostrum likely promote optimal calf health and development, potentially offering protective benefits against preweaning infection and disease. Yet, similar to IgG (Morrill et al., 2012), concentrations of bioactive compounds in bovine colostrum can range widely, with colostrum insulin (Malven et al., 1987; Aranda et al., 1991) and LF (Kehoe et al., 2007) exhibiting 55- and 22-fold differences, respectively, among animals. As the majority of US calves are fed maternal colostrum (Urie et al., 2018), it is necessary to identify dam factors that may impede calves from receiving “high-quality colostrum” to minimize their risk of disease. Particularly, we (Fischer-Tlustos et al., 2021) recently suggested that the definition of “colostrum quality” be expanded to include more components than simply IgG, emphasizing the need to determine potential associations between IgG, colostrum components (fat, protein, lactose) and bioactive compounds, which likely differ between individual dams or groups of animals. Such data are necessary to formulate a more complete definition of “colostrum quality” that ensures not only TPI, but also optimizes immune, gastrointestinal, and metabolic development of the neonatal calf.

There is currently a large body of evidence demonstrating differences in colostrum composition (Morrill et al., 2012) and bioactive compound (Ohtsuka et al., 2010; Fischer-Tlustos et al., 2020) concentrations between primiparous (**PP**) and multiparous (**MP**) cows, which may have a large impact on the development and metabolism of neonatal calves consuming colostrum from each respective parity. To the authors' knowledge, no studies have investigated the effect of parity on IGF-I in colostrum and transition milk (**TM**; milkings 2–6). Although both LF (Tsuji et al., 1990) and insulin (Zinicola and Bicalho, 2019) in colostrum may be influenced by parity, it is unclear if this is also true for TM.

It was hypothesized that parity would differentially affect the concentrations and yields of LF, insulin, and IGF-I in colostrum, TM, and WM, and that associations would be observed between colostrum and TM compounds, but that these associations would differ between MP and PP cows. Therefore, the objectives of the present study were to (1) determine the concentrations and yields of colostrum, TM, and WM insulin, IGF-I, and LF; and (2) determine their correlations with components and bioactive compounds in the first week of lactation in MP and PP cows.

All experimental procedures were conducted in accordance with the Canadian Council on Animal Care (1993) and were preapproved by the University of Alberta Animal Care and Use Committee for Livestock (AUP 00002015). The study presented herein is a companion article to Fischer-Tlustos et al. (2020), in which detailed methods regarding animal management, feeding, and sample collection are described. In brief, 10 MP and 10 PP healthy Holstein cows were enrolled in the animal experiment at Breevliet Farms Ltd. (Millet, AB, Canada). No cows enrolled in the study displayed clinical signs of illness or received antibiotics during the sampling period. The study location was selected due to its proximity to the University of Alberta, available resources (24/7 calving surveillance), and a herd size of ∼400 lactating cows, allowing for collection of all samples within a 60-d period to reduce seasonal variation. Although the sample size was determined for the original experimental objectives (Fischer-Tlustos et al., 2020), it was determined that the selected number of animals was sufficient to provide a power of 0.80 at a level of significance of 0.05 to detect a 10% difference in colostrum insulin (Mann et al., 2016) between parity.

Multiparous cows (parity = 3.1 ± 0.4 [mean ± SE]) were dried off at 53.4 ± 3.8 d relative to expected calving date. Primiparous cows were housed off-site until 3 wk prior to calving, after which MP and PP cows were group-housed and fed the same dry cow ration with a NE_L_ content of 1.31 Mcal/kg DM (12.7% CP, 18.4% starch, 48.7% NDF) until calving. After calving, MP and PP cows were fed the same lactating diet with a NE_L_ content of 1.57 Mcal/kg DM (15.7% CP, 24.9% starch, 32.5% NDF). Colostrum was collected at 5.3 ± 0.7 h after parturition, after which cows were milked at 0500 and 1700 h daily (time interval = 12.0 ± 0.3 h). Homogeneous samples of colostrum, milking (**M**) 2–5 (TM) and M12 (WM; d 6 postpartum) were collected from the milking system (Boumatic, Madison, WI). Yield was recorded and samples were immediately snap frozen in liquid nitrogen, then stored at −20°C until analysis.

Colostrum, TM, and WM of all cows (n = 20) were prepared for insulin analysis by skimming and diluting as described in Hare et al. (2023). Colostrum was diluted to 2% using double-distilled H_2_O, whereas subsequent milkings were diluted to 3% (M2) and 7% (M3 to M5). Whole milk (M12) was not diluted. Samples were analyzed using a bovine insulin ELISA kit (Mercodia, Uppsala, Sweden). Insulin plates were read at 450 nm using a BioTek Cytation 5 Imaging Reader (Agilent, Santa Clara, CA), and inter- and intra-assay CV were 4.7% and 4.6%, respectively.

The IGF-I concentrations (n = 20) were analyzed using an IGF-I ELISA kit (Mediagnost, Reutlingen, Germany). Samples were diluted with PBS at 1:40 (M1 and M2), 1:20 (M3 and M4), or 1:10 (M5 and M12). The concentration of LF in the same samples (n = 20) were analyzed using a Bovine Lactoferrin ELISA kit (Bethyl Laboratories Inc., Montgomery, TX). Samples for LF analysis were diluted with PBS at 1:10,000 (M1 and M2), 1:2,000 (M3 and M4), or 1:1,000 (M5 and M12). Both IGF-I and LF plates were read at 450 nm using Model 680 Microplate Reader (Bio-Rad Laboratories, Hercules, CA). The intra-assay and inter-assay CV were 5.8% and 8.6%, respectively, for the IGF-I ELISA and 8.5% and 9.8%, respectively, for the LF ELISA.

Statistical analysis was conducted using the GLIMMIX procedure of SAS Studio (version 9.4, SAS Institute Inc., Cary, NC). Datasets were analyzed for descriptive statistics and normality using the UNIVARIATE procedure. Using the SGPLOT procedure, residuals were confirmed to be random, homogeneous, and independent of treatment and design effects by visual inspection of distribution plots. When data did not follow a Gaussian distribution, outcome measurements were analyzed using the respective model with a lognormal distribution, after which LSM and 95% CI were back transformed and reported herein. For all outcome variables, the statistical model included the fixed effects of parity, milking, and the interaction of milking and parity, and cow was considered the experimental unit and a random effect. A Tukey test was used to separate LSM. For all data, at least 3 covariance structures for data with unequal spacing between sample collections were tested [cs, sp(gau), sp(pow)] and the one that yielded the lowest Akaike's information criterion [sp(pow)] was used. All values reported are LSM (95% CI). Significance was declared at *P* < 0.05 and tendencies at 0.10 < *P* < 0.05.

For each parity group, Spearman's rank correlation coefficients between colostrum and TM components (i.e., fat, protein, lactose, TS) and bioactive compounds from the current study and from a companion article (Fischer-Tlustos et al., 2020) were calculated in R (version 2022.07.2; R Core Team, 2023) with the *cor* function. Heatmaps were plotted using the ‘ggcorrplot' package (Kassambara, 2023). Correlation coefficients were defined as weak (|ρ| < 0.30), moderate (0.3 < |ρ| < 0.80), or strong (|ρ| > 0.80).

Results demonstrated that LF concentrations and yields were highest in colostrum and gradually decreased to M12 (Table 1). The mean colostrum LF concentration was markedly lower than those of Tsuji et al. (1990; 1,960 mg/L) and Kehoe et al. (2007; 820 mg/L) but was similar to values reported by Yoshida et al. (2000; 336 mg/L). Large individual variation was observed, with LF concentrations ranging from 97 to 5,631 mg/L. However, 85% of animals had LF concentrations <1,000 mg/L, with only MP cows having concentrations above this level. As such, mean LF concentrations and yields were increased by 59.7% (*P* = 0.0008; MP: 224.7 [159.3–316.9] mg/L; PP: 90.5 [64.2–127.7] mg/L) and 65.5% (*P* = 0.0002; MP: 2,252 [1,570.0–3,231.3] mg/milking; PP: 777.1 [541.6–1,115.0] mg/milking), respectively, in MP compared with PP cows over the sampling period. The MP cows had greater LF concentrations than PP cows in colostrum (*P* = 0.060), M2 (*P* = 0.049), and M3 (*P* = 0.022; Figure 1a). There was no difference between parity in colostrum LF yield (*P* = 0.90), but MP cows had greater LF yield in M2 (*P* = 0.064), M3 (*P* = 0.015), M5 (*P* = 0.043), and M12 (*P* = 0.003) than PP cows (Figure 1b). Tsuji et al. (1990) demonstrated similar findings, with MP cows producing colostrum with approximately 2 to 3 times greater LF concentrations than PP cows. We speculate that MP cows may possess greater mammary LF to combat higher rates of subclinical IMI during the dry period (Dahl et al., 2020), when the majority of IMI begin (Green et al., 2002). It is also possible that the high colostrum LF concentrations observed in MP cows may be related to the process of regenerative involution, given high mRNA levels after milking cessation (Wilde et al., 1997) and the ability of LF to remodel the extracellular matrix in vitro (Nakajima et al., 2015). As such, LF may accumulate in the stromal areas of MP cows during involution, creating a pool for secretion into colostrum during its synthesis.

**Table 1:** Insulin, IGF-I, and lactoferrin concentrations and yields in colostrum (milking 1), and milkings 2–5 and 12 in multiparous (n = 10) and primiparous (n = 10) Holstein dairy cows milked twice daily^1^

Parameter	Milking						*P*-value
	1	2	3	4	5	12	
Concentration							
Insulin (μg/L)	21.2^a^	8.7^b^	2.8^c^	1.2^d^	1.4^d^	0.5^e^	<0.0001
	(14.8–30.3)	(6.1–12.4)	(2.0–4.0)	(0.9–1.8)	(1.0–2.0)	(0.3–0.7)	
IGF-I (μg/L)	479.9^a^	205.2^b^	124.8^c^	69.8^d^	30.8^e^	13.2^f^	<0.0001
	(378.9–608.0)	(162.0–260.0)	(98.5–158.1)	(55.1–88.4)	(24.3–39.0)	(10.4–16.7)	
Lactoferrin (mg/L)	385.4^a^	183.0^b^	160.4^b^	117.7^c^	95.1^cd^	66.4^d^	<0.0001
	(286.4–518.5)	(136.0–246.3)	(119.2–215.8)	(87.5–158.4)	(70.7–128.0)	(49.4–89.4)	
Yield							
Insulin (μg)	105.2^a^	59.3^ab^	25.6^bc^	14.8^cd^	19.5^d^	5.9^d^	<0.0001
	(68.9–160.6)	(38.6–91.0)	(16.8–39.0)	(9.7–22.7)	(12.8–29.8)	(3.9–9.0)	
IGF-I (μg)	2,384.0^a^	1,432.0^b^	1,137.9^bc^	835.8^c^	420.1^d^	162.4^e^	<0.0001
	(1,807.4–3,144.6)	(1,082.0–1,894.4)	(862.7–1,500.9)	(633.6–1,102.4)	(318.5–554.2)	(123.1–214.2)	
Lactoferrin (mg)	1,914.2^a^	1,282.9^b^	1,462.4^ab^	1,410.6^ab^	1,296.9^ab^	816.2^b^	0.0003
	(1,377.7–2,659.8)	(921.7–1,785.7)	(1,052.5–2,032.0)	(1,015.2–1,959.9)	(933.3–1,802.0)	(587.4–1,134.0)	

a–f Different letters represent differences (*P* < 0.05) between milkings.

1 Values represent LSM (95% CI).

**Figure 1:**
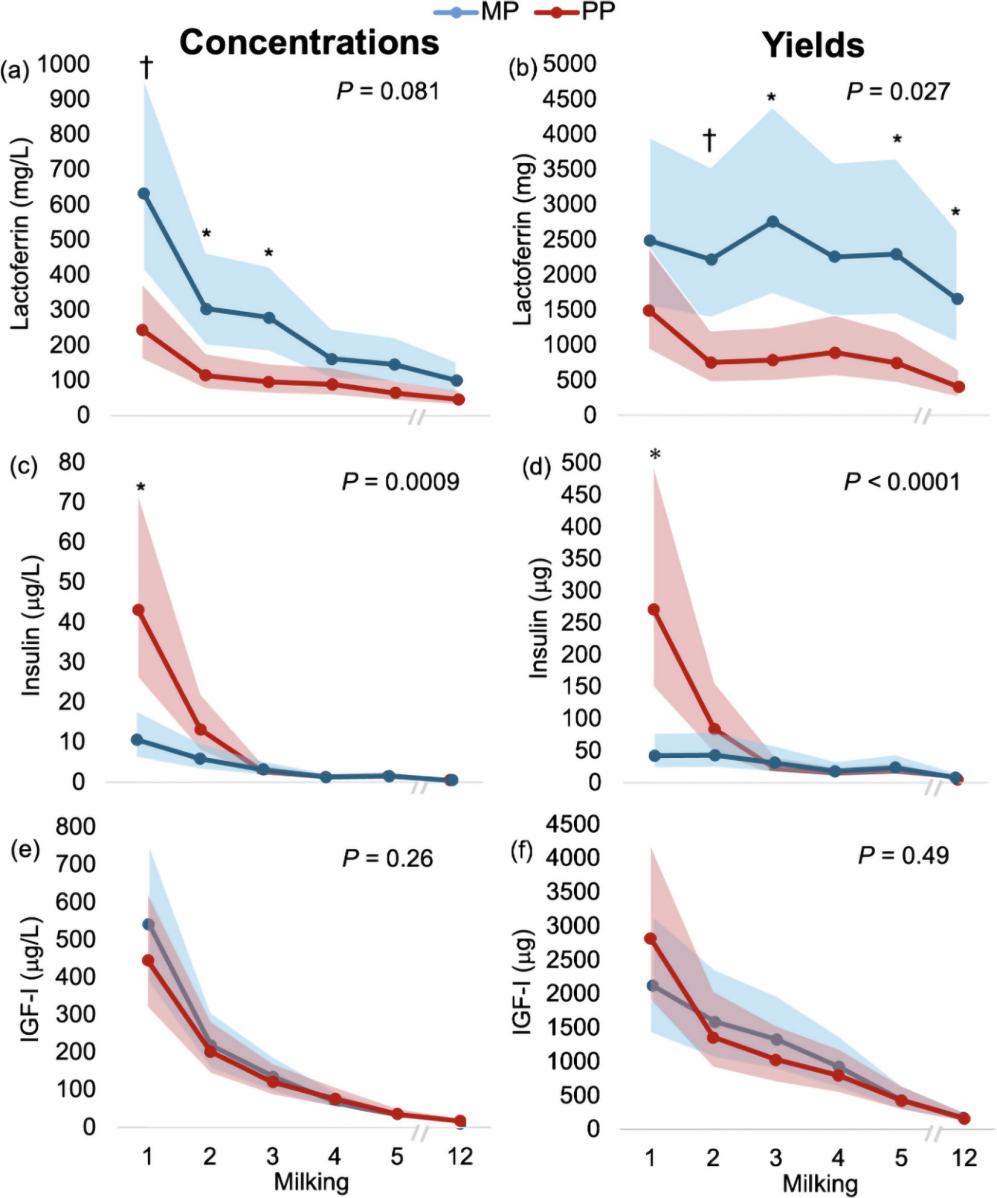
Concentrations and yields of lactoferrin (a, b), insulin (c, d), and IGF-I (e, f) in colostrum (milking 1), and milkings 2–5 and 12 in multiparous (MP [blue]; n = 10) and primiparous (PP [red]; n = 10) Holstein dairy cows milked twice daily. Points represent LSM ± 95% CI. A * and † represent a significant difference (*P* < 0.05) and tendency (0.05 < *P* < 0.10), respectively, between MP and PP cows within milking (parity × milking).

Mean insulin concentration in colostrum was 21.2 (14.8–30.3) μg/L (Table 1), which is lower than insulin concentrations reported by Zinicola and Bicalho (2019; 106 μg/L for PP cows; 54 μg/L for MP cows), Aranda et al. (1991; 327 μg/L), and Blum and Hammon (2000; 64 μg/L). The low mean colostrum insulin concentration appeared to be substantially influenced by MP cows, as all MP cows had colostrum insulin concentrations <30 μg/L, whereas only 2 PP cows had colostrum insulin concentrations <30 μg/L. This led to PP cows having 4 and 6 times greater colostrum insulin concentrations and yields, respectively, compared with MP cows (Figure 1c, d), which is in line with results reported by Zinicola and Bicalho (2019). Mammary uptake of insulin may occur via active transport, with the greatest mammary uptake of insulin occurring at 1 to 3 d prior to calving (Malven et al., 1987) as demonstrated by a positive correlation between plasma insulin during this timeframe with colostrum insulin (Mann et al., 2016). If colostrum insulin is proportional to plasma insulin prior to calving, then higher prepartum circulating insulin concentrations in PP cows compared with MP cows (Janovick et al., 2011) may account for this finding.

In contrast to LF and insulin, IGF-I concentrations (*P* = 0.88) and yields (*P* = 0.49) did not differ by parity within specific milkings (Figure 1e, f), but concentrations were similar to those reported by Samanc et al. (2009; 482 μg/L) and Nikolic and Masnikosa (1998; 345 μg/L). Interestingly, insulin and IGF-I concentrations and yields were higher in colostrum compared with WM, being 43.9 and 17.8 times greater for insulin and 36.3 and 14.7 times greater for IGF-I, respectively, whereas LF concentrations and yields were only 5.8 and 2.3 times greater, respectively. The reason behind the relatively lesser decrease in LF from the colostral and TM period to WM, compared with the more pronounced reductions observed in insulin and IGF-I, as well as other bioactive compounds (Blum and Hammon, 2000; Fischer-Tlustos et al., 2020), remains unknown and requires additional research.

At present, IgG content is the key metric for evaluating “colostrum quality,” despite the estimated hundreds of colostral bioactive compounds that may be crucial for neonatal development and metabolism. As such, this study aimed to determine potential associations between commonly measured colostrum compounds and components (i.e., fat, protein, lactose, TS), IgG, and bioactive compounds. All strong (ρ > 0.80) correlations in colostrum of both MP (Figure 2a) and PP (Figure 2b) were positive. However, positive correlations between IgG yield and milk (ρ = 0.98), fat (ρ = 0.75), lactose (ρ = 0.78), and TS (ρ = 0.94) yield were uniquely observed in PP cows, potentially indicating that colostrum IgG accumulation may occur simultaneously with mammary epithelial cell development and component synthesis in PP cows. Between bioactive components, PP cows only showed associations between IgG and IGF-I (ρ = 0.83), and IgG and LF (ρ = 0.64). Correlations existed among other bioactive compounds in PP colostrum but were not as strong (ρ < 0.60). In the colostrum of MP, but not PP, cows, insulin was positively correlated with milk (ρ = 0.62), protein (ρ = 0.67), TS (ρ = 0.58), IgG (ρ = 0.55), and total OS (ρ = 0.71) yield. Overall, it appeared that colostrum of MP cows demonstrated a greater number of correlations among bioactive compounds (IgG, IGF-I, insulin, OS), whereas PP cows demonstrated a greater number of correlations between components and bioactive compounds.

**Figure 2:**
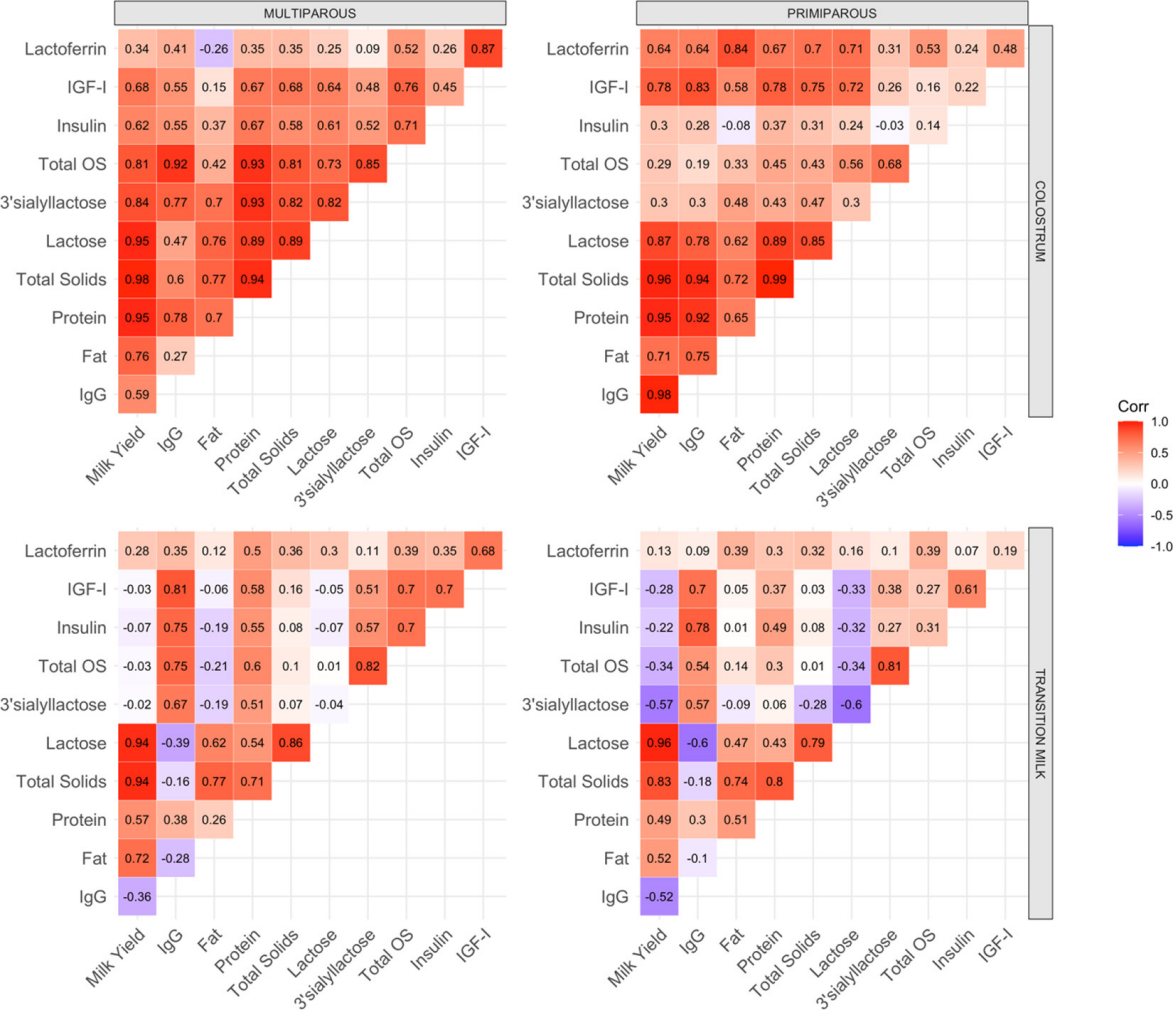
Spearman's rank correlation coefficients between composition (fat, protein, lactose, TS) yields, total oligosaccharide (OS) and 3′sialyllactose yields, IgG yield, and milk yield (Fischer-Tlustos et al., 2020) and the bioactive peptide yields reported herein (insulin, IGF-I, lactoferrin) in colostrum (milking 1) and transition milk (milkings 2 to 5) of multiparous and primiparous cows. Correlation (Corr) coefficients range from positive (ρ = 1.0; red) to negative (ρ = −1.0; blue).

In contrast to colostrum, TM generally contains low concentrations of IgG but elevated concentrations of other components and bioactive compounds (Blum and Hammon, 2000). For this reason, we evaluated the associations between components and bioactive compounds over the TM period (Figure 2c, d). In both MP and PP cows, IgG was negatively correlated with lactose and milk yield; however, these correlations were stronger in PP than MP cows. As expected, milk yield was positively correlated with lactose, TS, and fat yield in both parity groups. In TM, insulin and IGF-I were both positively (ρ > 0.70) correlated with IgG and total OS in MP cows, whereas insulin and IGF-I was only correlated to the same magnitude (ρ > 0.70) with IgG, and not total OS, in PP cows. Lactose was negatively correlated with insulin, IGF-I, total OS, and 3′sialyllactose yield of PP cows only. It is particularly interesting that lactose was only negatively correlated to total OS in PP, and not MP, cows. This may suggest that high rates of synthesis and export of lactose may only negatively influence the synthesis of OS in TM of PP cows, but not MP cows. This finding may potentially explain higher concentration and yield of certain OS, namely 6′sialyllactosamine, in TM of MP compared with PP cows (Fischer-Tlustos et al., 2020). It is important to note that although the presented associations are noteworthy, they were conducted with low replication per parity and further work utilizing a larger dataset is necessary to confirm the results presented above.

In conclusion, our study demonstrated a rapid decline in IGF-I and insulin, and a more gradual decline in LF, from colostrum to WM. In addition, parity influenced early lactation insulin and LF, but had no effect on IGF-I. As such, results highlight differences in colostrum and TM composition between PP and MP cows, which may influence the physiology and development of neonatal calves consuming these secretions from each respective parity. Correlation analyses highlighted potential differences in mammary synthesis upon initiation of first lactation compared with mammary remodeling in MP cows. Specifically, PP cows had a greater number of strong correlations between early lactation components and bioactive compounds than MP cows, which primarily displayed correlations between bioactive compounds.
